# A suitable palpation technique allows to identify skin lipohypertrophic lesions in insulin-treated people with diabetes

**DOI:** 10.1186/s40064-016-1978-y

**Published:** 2016-05-05

**Authors:** Sandro Gentile, Giuseppina Guarino, Annalisa Giancaterini, Piero Guida, Felice Strollo, AMD-OSDI Italian Injection Technique Study Group

**Affiliations:** Department of Internal Medicine, Second University of Naples, Naples, Italy; Head of the Medical Branch, Outpatient Care Network, Milan, Italy; Statistical Consultant for Associazione Medici Diabetologi (AMD), Rome, Italy; Diabetes and Endocrinology, Elle-di, Via degli Scipioni 175, 00192 Rome, Italy

**Keywords:** Diabetes, Lesions, Lipohypertrophy, Insulin, Injection

## Abstract

**Background:**

Lipohypertrophy (LH) is a major complication of subcutaneous insulin treatment brought about by multiple overlapping injections and/or needle reuse. It is responsible for unacceptable glucose oscillations due to a high rate of hypoglycaemic episodes and rebound glucose spikes. Skin ultrasound scans (USS), the gold standard for its detection, is too expensive for screening purposes.

**Aims:**

To define a structured method allowing health professionals (HPs) to identify LH lesions as inexpensively and correctly as possible.

**Methods:**

Out of 129 insulin-treated people with diabetes identified by USS as having LH lesions, only 40 agreed to participate in the study (24 females, age 54 ± 15 years, daily insulin dosage 57 ± 12 IU). Each was blindly examined by four well trained and four non-trained HPs according to a standard method involving repeated well codified maneuvers.

**Results:**

A specific training allowed inexperienced HPs to acquire high diagnostic accuracy in identifying LH lesions independent of site, size, shape, and even BMI. This kind of training also allowed to reach a 97 % consistency rate among HPs as compared to USS, while the lack of training was associated with a wide variability and inconsistency of identification results.

**Conclusions:**

Diabetes teams should follow systematically the simple procedure reported in this paper for the diagnosis of LH and try to get it further implemented and progressively refined in large scale studies. This would have a major impact on patient education in terms of (1) correct injection technique and (2) ability to identify lesions early enough to prevent poor metabolic outcome.

## Background

An appropriate injection technique is essential for optimal insulin effect in diabetes mellitus (DM) (Frid et al. 2010). Lipodystrophy, including lipohypertrophy (LH) and lipoatrophy (LA), is a major complication of subcutaneous insulin shots, LH being by far more frequent than LA.

LH presents itself as a thickened, ‘rubbery’ tissue swelling caused by typical growth-enhancing insulin properties associated with skin reaction due to multiple overlapping injections and/or needle reuse (Thow et al. 1990; Richardson and Kerr 2003). Most studies suggest that insulin absorption from sites characterized by LH may be both delayed and erratic. As a consequence of that, ever increasing doses of insulin are required and metabolic control gets worst (Young et al. 1984; Frid and Linden 1992; Chowdhury and Escudier 2003; Johansson et al. 2005; Gentile et al. 2011). This in turn causes unacceptable glucose oscillations due to a high rate of serious hypoglycaemic episodes followed by rebound glucose spikes in the cases of patients suddenly switching from altered to normal injection sites. All this has also a strong impact on the economic burden of the disease for both patients and health care system.

Therefore, it is crucial to try and systematically identify as many LH areas as possible in order to educate patients to prevent poor insulin injection habits.

Papers published on this topic so far show great differences in their prevalence in insulin treated patients (see Table 1). This is probably due to the lack of a well-structured diagnostic flow-chart despite the world-wide availability of suitable ultrasound and radiological methods (Seyoum and Abdulkadir 1996; Hauner et al. 1996; McNally et al. 1988; Partanen and Rissanen 2000; Raile et al. 2001; Kordonouri et al. 2002; Teft 2002; Vardar and Kizilci 2007; Hajheydari et al. 2011; Blanco et al. 2013; Grassi et al. 2014).

**Table 1:** Lipohyperthophy prevalence variability among different case studies

	Publication year	Prevalence (%)	Author	Diabetes type 1 or 2
Seyoum	1996	31.0	9	1 + 2
Hauner	1996	28.7	10	1
Partanen	2000	34.5	11	1
Raile	2001	27.1	12	1
Kordonouri	2002	48.0	13	1
Vardar	2007	48.8	14	1 + 2
Hajheydari	2011	14.5	15	1 + 2
Teft	2002	57.0	16	1 + 2
Blanco	2013	64.0	17	1 + 2
Grassi	2014	49.0	18	1 + 2
McNally	1988	28.0	19	2
Hauner	1996	3.6	10	2

Based on these premises, we compared well-trained vs untrained health care providers in terms of their ability to identify LH lesions of different type, site and size in people with diabetes selected by an experienced physician and precisely characterized by ultrasound scans (USS).

The final goal of this study was to define a structured inexpensive method allowing health professionals (HPs) to identify LH lesions as easily and correctly as possible during routine examinations.

## Methods

### Ethical aspects

The protocol was prepared according to the Helsinki declaration and approved by the local Ethics Committee.

### Consent to publish

Consent to publish was obtained from the participants to report their individual data.

### Subjects

All patients gave their informed consent for participation in the research study.

An experienced physician referring to our clinic examined 265 people with DM (59 with type 1 DM) who had been on insulin for more than one year with at least three injections a day of rapid and basal analogues using prefilled pens with 5 mm/31 G needles. Patients on human insulin or NPH, as well as, those using needles of different length and thickness were intentionally excluded from the survey in order to rule out any possible confounding factors, because other groups already showed that needle length and gauge correlate with the risk of LH as well as with the quality of metabolic control (Frid et al. 2010; Kreugel et al. 2011; Hansen and Matytsina 2011; Blanco et al. 2013).

Out of these 265, 129 people were identified as having LH lesions (48.8 %) at one or more injection sites but only 40 (45.9 %) agreed to participate in the study. The main clinical features of participants are given in Table 2 and may be briefly summarized as follows: 24 were females, age was 54 ± 15 years, daily insulin dosage was 57 ± 12 IU, all followed a four daily shot regimen.

**Table 2:** Patient features by lipohypertrophy (LH) site, shape and size

	Overall	Site			Shape		Diameter	
		Abdomen	Arm	Thigh	Flat	Protruding	≤4 cm	>4 cm
	n = 40	n = 16	n = 14	n = 10	n = 22	n = 18	n = 20	n = 20
Female gender	60 %	12 (75 %)	8 (57 %)	4 (40 %)	16 (72.7 %)	8 (44.4 %)	10 (50 %)	14 (70 %)
BMI (kg/m^2^)	29.1 ± 2.4	28.8 ± 3.2	29.7 ± 2.3	28.5 ± 1.2	29 ± 2.1	29.1 ± 2.9	28.8 ± 1.8	29.4 ± 3
LH site								
Abdomen	16 (40 %)	16 (100 %)	0 (0 %)	0 (0 %)	*4 (18.2 %)*	*12 (66.7 %)*	*2 (10 %)*	*14 (70 %)*
Arm	14 (35 %)	0 (0 %)	14 (100 %)	0 (0 %)	*14 (63.6 %)*	*0 (0 %)*	*10 (50 %)*	*4 (20 %)*
Thigh	19 (25 %)	0 (0 %)	0 (0 %)	10 (100 %)	*4 (18.2 %)*	*6 (33.3 %)*	*8 (40 %)*	*2 (10 %)*
LH shape								
Flat	22 (55 %)	*4 (25 %)*	*14 (100 %)*	*4 (40 %)*	22 (100 %)	0 (0 %)	16 (80 %)	6 (30 %)
Protruding	18 (45 %)	*12 (75 %)*	*0 (0 %)*	*6 (60 %)*	0 (0 %)	18 (100 %)	4 (20 %)	14 (70 %)
LH size								
Diameter (cm)	4.8 ± 1.5	*10.6 ± 1.2*	*4.0 ± 1.4*	*4.8 ± 1.8*	*4.2 ± 1.3*	*5.7 ± 1.5*	3.6 ± 0.5	6.1 ± 1.1
Diameter ≤ 4 cm	20 (50 %)	*2 (12 %)*	*10 (71 %)*	*8 (80 %)*	16 (72.7 %)	4 (22.2 %)	20 (100 %)	0 (0 %)
Diameter > 4 cm	20 (50 %)	*14 (88 %)*	*4 (29 %)*	*2 (20 %)*	6 (27.3 %)	14 (77.8 %)	0 (0 %)	20 (100 %)

Mean ± standard deviation or rate (percentage). Italics characters are used for statistically significant comparisons (*p* < 0.05)

### Methodological aspects of high-frequency skin ultrasound scans

Skin USS were performed at all participants’ injection sites to validate the diagnosis of LH and to define single lesion features, including size, thickness and texture. USS were repeated by different operators on the same patient according to the procedure described in the methodology section.

High-frequency B-mode skin USS were performed invariably using the linear 20 MHz probe (Philips HD3). Each specialist performed five consecutive scans of each possible site on one day to assess intra-operator variation (IntraOV) and on three consecutive days to assess day-to-day operator variation (D-T-DOV). Moreover three different operators performed their scans at 2 h intervals between 08:00 and 20:00 h to assess inter-operator variation (InterOV).

A 100 % consistency in LH identification was found among specialists for IntraOV, InterOV and D-T-DOV, independently of location, volume, extension, texture or thickness. This result confirmed USS to be a gold standard method for subsequent palpation results.

### Study protocol

After being instructed to refrain from revealing their LH areas to anyone, all patients were examined by four non-trained (NT) and four well trained (WT) HPs.

NT HPs were given no advice on how to inspect and touch the skin and were simply asked to repeatedly try and identify lesions in each expected location by examining the injection sites the best they could.

On the opposite, WT HPs were taught how to correctly define LH lesions by performing a careful examination of typical injection sites according to the protocol described below.

### Lesion identification training protocol

**Fig. 1:**
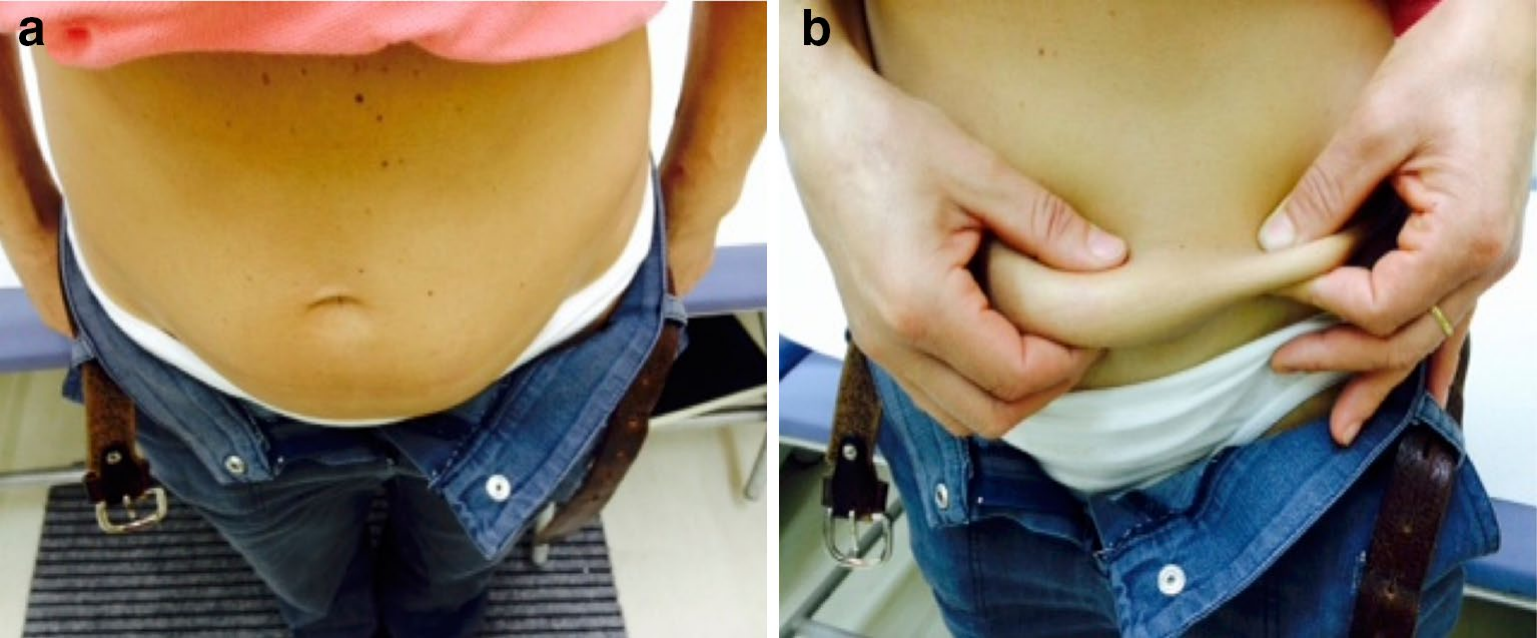
Lipohypertrophy features. Moderate swelling of the abdominal wall below the umbilicus, the site most often chosen by the patient for insulin injections; his right hand pinches a thick fold in the presence of a large lipohypertrophy skin plate (**a**); while only a thin fold results from the left hand squeezing the area systematically ignored for insulin shots (**b**)

**Fig. 2:**
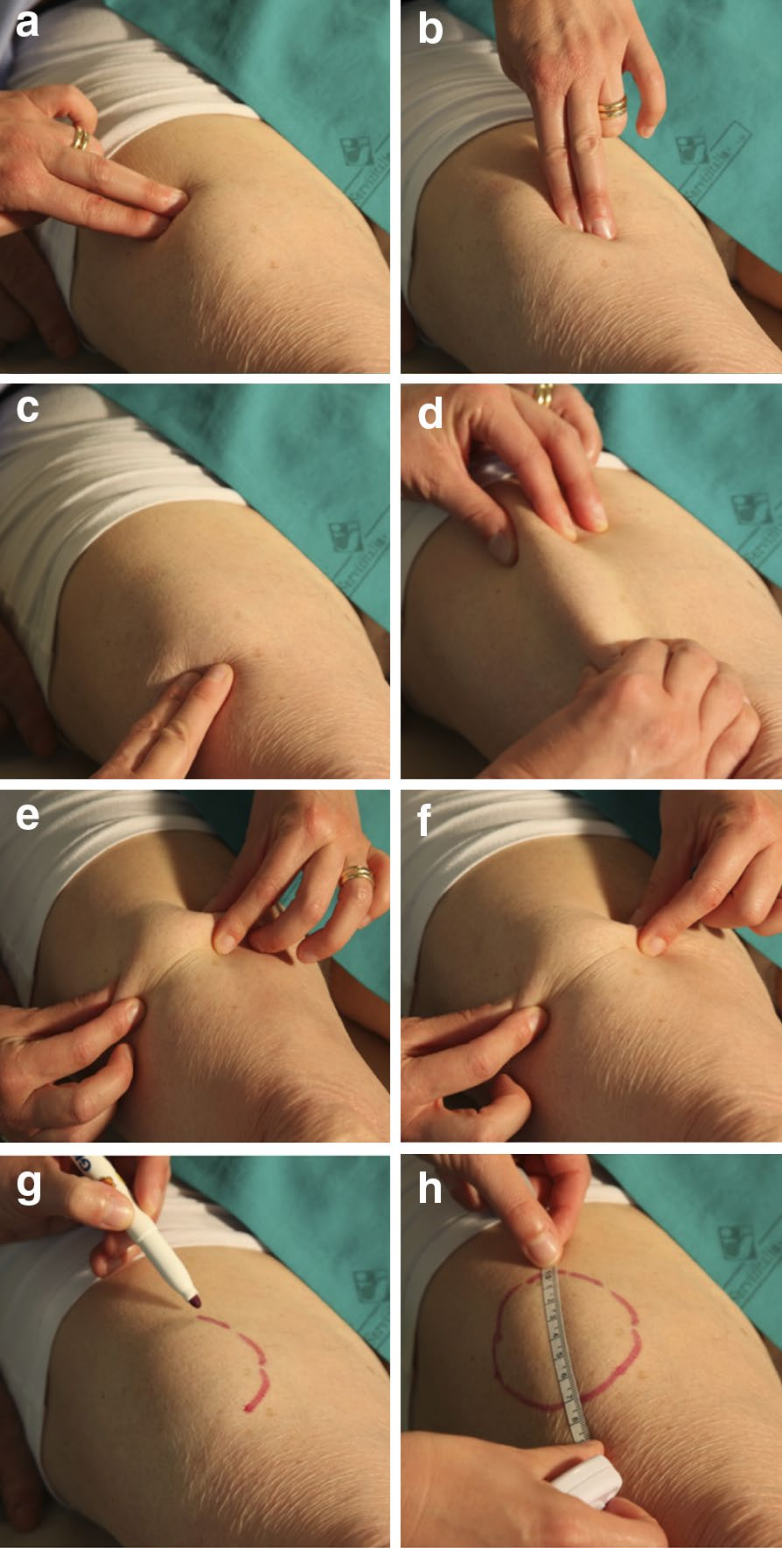
Lipohyertrophy identification technique. The figure shows how to identify a LH lesion after a thorough inspection of the area by performing repeated vertical and horizontal finger tip movements over and around it (**a**–**c**), pinching it (**d**–**f**) and marking it (**g**) and how to finally measure it (**h**)

The method consisted of the inspection of each interested area using direct and tangential light against a dark background, as well as, of a thorough palpation technique (slow circular and vertical finger tip movements followed by repeated horizontal attempts on the same spot). HPs were also advised to be gentle while touching the skin at the beginning and start to progressively increase finger pressure thereafter. They were also suggested to perform the pinch maneuver when perceiving a harder skin, to confirm their first impression by comparing the thickness of the suspected spot to that of surrounding areas (Fig. 1). Smaller and flatter lesions were best identified by repeating all above mentioned palpation maneuvers (Fig. 2).

### Statistical analysis

The continuous variables were reported as mean value ± Standard Deviation. Between-group comparisons made use of the Mann–Whitney test. Categorical variables were summarized in terms of frequency and percentage and the Fisher test was used to evaluate associations among them. The data repeatedly recorded by WT and NT groups were analyzed according to a mixed logistic model with patients fitted as random. The mixed model provided separate estimates of the proportion of patients correctly identified by WT and NT health professionals. The association between patients characteristics and missed LH identification was evaluated by means of univariate and multivariate logistic mixed models. Odds ratios (ORs) were given with their 95 % confidence intervals (CIs). All analyses were carried out using STATA software, Version 12 (Stata Corp, College Station, TX, USA) with *p* values <0.05 a priori accepted as significant.

## Results

As described in Table 2, LH lesions were found in 16 patients on the abdomen, in 14 on the arms and in 10 on the thighs. 22 lesions were flat and 18 were protruding; in 20 the lesion diameter was >4 cm. By analyzing the data, a relationship could be found between LH location, shape and size, where the smallest lesions were mostly flat and located on the arms. Figure 1 shows the appearance of large abdominal LH lesions in an overweight patient.

Table 3 shows the results provided by the two groups of HPs analyzed with respect to location, thickness and size. NT HPs were different in their ability to identify LH, thus displaying a diagnostic sensitivity of 72 % (51–87 %) vs the 96 % (89–99 %) found in WT HPs.

**Table 3:** Lipohypertrophy identification rate for Well Trained (WT) and Non-Trained (NT) health professionals by site, shape and size

	Overall	Site			Shape		Diameter	
		Abdomen	Arm	Thigh	Flat	Protruding	≤4 cm	>4 cm
	n = 40	n = 16	n = 14	n = 10	n = 22	n = 18	n = 20	n = 20
WT								
1	40 (100 %)	16 (100 %)	14 (100 %)	10 (100 %)	22 (100 %)	18 (100 %)	20 (100 %)	20 (100 %)
2	36 (90 %)	16 (100 %)	10 (71 %)	10 (100 %)	18 (82 %)	18 (100 %)	18 (90 %)	18 (90 %)
3	38 (95 %)	16 (100 %)	12 (86 %)	10 (100 %)	20 (91 %)	18 (100 %)	18 (90 %)	20 (100 %)
4	40 (100 %)	16 (100 %)	14 (100 %)	10 (100 %)	22 (100 %)	18 (100 %)	20 (100 %)	20 (100 %)
NT								
1	28 (70 %)	14 (88 %)	4 (29 %)	10 (100 %)	10 (46 %)	18 (100 %)	10 (50 %)	18 (90 %)
2	24 (60 %)	14 (88 %)	4 (29 %)	6 (60 %)	10 (46 %)	14 (78 %)	8 (40 %)	16 (80 %)
3	32 (80 %)	12 (75 %)	10 (71 %)	10 (100 %)	18 (82 %)	14 (78 %)	16 (80 %)	16 (80 %)
4	22 (55 %)	14 (88 %)	4 (29 %)	4 (40 %)	8 (36 %)	14 (78 %)	6 (30 %)	16 (80 %)

**Fig. 3:**
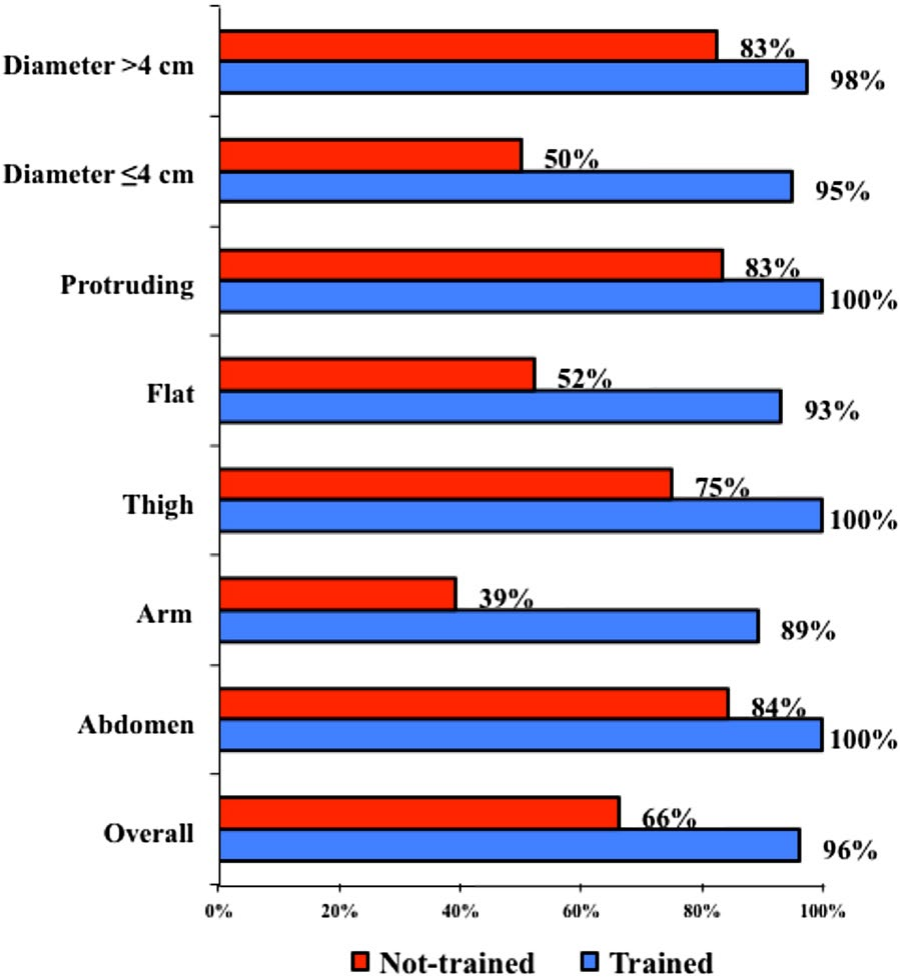
Lipohypertrophy identification results obtained by trained and non-trained health professionals as referred to the shape, site and size of skin lesions (% stays for identification rate)

Figure 3 summarizes the results of the two groups and shows lower percentage of correct classifications in the NT group in the presence of smaller or flatter lesions, especially those occurring at the arm level.

Table 4 shows parameters related to missed LH identification. The top half of the table describes the analysis of data recorded by NT HPs, and the bottom half refers to the whole population under study: a significantly higher risk of missing LH identification was shown by the NT group. The factors associated to missed identification were again small size, flat shape and arm location while BMI did not affect the results.

**Table 4:** Univariate and multivariate predictors of missed lipohypertrophy (LH) identification by well trained (WT) and non-trained (NT) health professionals (HPs)

	Univariate	p	Multivariate	p
NT				
Female gender	1.91 (0.32–11.50)	0.480		
BMI (Kg/m^2^)	1.29 (0.89–1.87)	0.172	–	
LH site				
Abdomen	1.00		–	
Arm	*10.75 (2.11–54.63)*	*0.004*	*6.04 (1.11–32.79)*	*0.037*
Thigh	1.90 (0.34–10.67)	0.467	–	0.999
LH shape				
Protruding	1.00		–	
Flat	*6.27 (1.25–31.62)*	*0.026*	0.84 (0.14–5.20)	0.855
LH size				
Diameter ≤4 cm	*6.90 (1.34–35.41)*	*0.021*	3.78 (0.92–15.58)	0.066
Overall				
NT versus WT	*24.87 (5.69–108.74)*	*<0.001*	*24.27 (5.58–105.58)*	*<0.001*
Female gender	1.88 (0.31–11.44)	0.492	–	
BMI (Kg/m^2^)	1.31 (0.91–1.89)	0.150	–	
LH site				
Abdomen	1.00		–	
Arm	*12.49 (2.65–58.92)*	*0.001*	*7.20 (1.35–38.36)*	*0.021*
Thigh	1.86 (0.34–10.18)	0.475	–	
LH shape				
Protruding	1.00		–	
Flat	*7.11 (1.43–35.29)*	*0.016*	0.92 (0.14–5.84)	0.930
LH size				
Diameter ≤4 cm	*6.63 (1.24–35.50)*	*0.027*	3.17 (0.78–12.81)	0.106

Univariate analysis in the overall group was performed after adjusting for HPs’ experience (NT o WT)

## Conclusions

An ever increasing number of reports in the literature points to a poor insulin administration technique as the main cause of skin LH (Thow et al. 1990; Frid and Linden 1992; Seyoum and Abdulkadir 1996; Hauner et al. 1996; Raile et al. 2001; Kordonouri et al. 2002; Vardar and Kizilci 2007; Hajheydari et al. 2011) and of their metabolic consequences, including wide blood glucose variability, as well as, severe unexplained hypoglycemic episodes (Young et al. 1984; Chowdhury and Escudier 2003; Richardson and Kerr 2003; Johansson et al. 2005; Gentile et al. 2011). Many papers also provide recommendations on how to properly inject insulin (Frid et al. 2010). Despite this, the extremely wide variation in LH rate reported so far in insulin-treated patients (Hauner et al. 1996; McNally et al. 1988; Partanen and Rissanen 2000; Raile et al. 2001; Kordonouri et al. 2002; Teft 2002; Vardar and Kizilci 2007; Hajheydari et al. 2011; Blanco et al. 2013; Grassi et al. 2014) proves that no systematic educational program has been implemented world-wide to teach people with diabetes how to correctly inject insulin (Blanco et al. 2013; Grassi et al. 2014). It also reflects the fact that the literature is still lacking a clear, explicit and standardized methodology describing how to recognize and diagnose LH lesions regardless of size, location and texture (Seyoum and Abdulkadir 1996; Hauner et al. 1996; McNally et al. 1988; Partanen and Rissanen 2000; Raile et al. 2001; Kordonouri et al. 2002; Teft 2002; Vardar and Kizilci 2007; Hajheydari et al. 2011; Blanco et al. 2013; Grassi et al. 2014). Moreover, despite their fully accepted role in the diagnosis of LH, USS are too costly to be proposed for routinely clinical examinations (Bianchi and Martinolli 2007; Lo Presti et al. 2012; Perciun and Mihu 2014).

We were able to show for the first time that a specific training involving repeated and well codified maneuvers made inexperienced HPs easily acquire diagnostic accuracy in identifying LH lesions independent of site, size, shape, and even BMI. This kind of training also granted a 97 % consistency rate among HPs as compared to the gold standard represented by skin USS, while the lack of training was associated with a wide variability and inconsistency of identification results. Therefore, we feel like interpreting the extremely wide variability in LH frequency reported by the literature as the consequence of the lack of a clear definition of suitable procedures for lesion identification.

An apparent limitation of our study was the relatively small number of patients and HPs involved. Nevertheless, our sample size was in line with that of many others reported in the literature so far as referred to highly specific endpoints. Moreover it provided statistically significant results, thus proving to be large enough for our aim (i.e. merely to help identify a straightforward solution for a clinically relevant problem).

In conclusion, we propose diabetes teams to follow systematically the simple above-reported procedure for the diagnosis of LH at all insulin shot sites and to get it further implemented and hopefully progressively refined in large scale studies.

This would have a major impact for patient education as WT health professionals might (1) verify whether or not their patients inject insulin correctly and, even better, (2) make patients really aware of the importance of their injection technique and eager to learn how to identify their own lesions early enough to prevent poor metabolic control (Polak et al. 1996; EADV 2008; Heinemann 2010).
